# Supplementary material for: Development of a computer-aided design software for the quantitative evaluation of aesthetic damage
Source: PLoS One. 2019 Dec 18;14(12):e0226322. doi: 10.1371/journal.pone.0226322 (PMC6919621; doi:10.1371/journal.pone.0226322)
Supplement: S1 Code site — 1. (ZIP) [file pone.0226322.s001.zip › [EESC_jr][Projeto]Relatorio_07_21/vendors/select2/docs/_includes/examples/hide-search.html]

# Hiding the search box

Select2 allows you to hide the search box depending on the number of
options which are displayed. In this example, we use the value
`Infinity` to tell Select2 to never display the search box.

{% highlight js linenos %}
$(".js-example-basic-hide-search").select2({
minimumResultsForSearch: Infinity
});
{% endhighlight %}
